# Self- and Other-Orientation in High Rank: A Cultural Psychological Approach to Social Hierarchy

**DOI:** 10.1177/10888683231172252

**Published:** 2023-05-25

**Authors:** Matthias S. Gobel, Yuri Miyamoto

**Affiliations:** 1 University of Sussex, Brighton, UK; 2 Hitotsubashi University, Tokyo, Japan

**Keywords:** social hierarchy, power, culture, interdependence, interpersonal processes, social cognition, organizational behavior

## Abstract

Social hierarchy is one fundamental aspect of social living, structuring interactions in families, teams, and entire societies. In this review, we put forward a cultural psychological approach to social hierarchy, positing that rank differences are embedded within larger cultural meaning systems, which shape how high rank is attained or conferred and how social hierarchy affords behavior and psychology. We then examine manifestations of hierarchy in two cultural meaning systems: Western and East Asian cultural contexts. Accumulating evidence on collective, interpersonal, and individual processes suggests cultural similarities in self-orientation but cultural differences in other-orientation of high-ranking individuals. Such literature reveal how thought and behavior within social hierarchies and cultural beliefs, values, and norms mutually constitute each other. We close with a discussion of how the present review is a stepping stone for future research and of remaining questions to further advance social hierarchy research across wider and more diverse cultural contexts.

**Public Abstract:**

Social hierarchy is one fundamental aspect of human life, structuring interactions in families, teams, and entire societies. In this review, we put forward a new theory about how social hierarchy is shaped by the wider societal contexts (i.e., cultures). Comparing East Asian and Western cultural contexts, we show how culture comprises societal beliefs about who can raise to high rank (e.g., become a leader), shapes interactions between high- and low-ranking individuals (e.g., in a team), and influences human thought and behavior in social hierarchies. Overall, we find cultural similarities, in that high-ranking individuals are agentic and self-oriented in both cultural contexts. But we also find important cross-cultural differences. In East Asian cultural contexts, high-ranking individuals are also other oriented; they are also concerned about the people around them and their relationships. We close with a call to action, suggesting studying social hierarchies in more diverse cultural contexts.

*“Business opportunity seldom knocks on the door of self-centered people”*

—Kazuo Inamori

*“Don’t let the noise of others’ opinions drown out your inner voice”*

—Steve Jobs

Social hierarchy is one of the most important features of social living. It fundamentally organizes interpersonal interactions (S. T. Fiske, 2010; A. P. Fiske et al., 1998; Van Vugt, 2006). For example, social hierarchy enables collective locomotion (Blau, 1964; Thibaut & Kelley, 1959) and increases team performance (Anicich et al., 2015; Halevy et al., 2012; Ronay et al., 2012). When a clear hierarchical structure is missing, group performance can suffer (Bendersky & Hays, 2012; Greer et al., 2011; Greer & van Kleef, 2010). Importantly, being of higher rank offers advantageous life outcomes. For example, high-ranking primates will get groomed more frequently (Seyfarth, 1977), and they can procreate more (Boehm, 1999; de Waal, 1989). Similarly, high-ranking humans receive greater respect and admiration from others (Berger et al., 1972; Ridgeway & Cornell, 2006), and they have greater access to valued resources (Van Vugt et al., 2008; Willer, 2009). Indeed, high rank reduces stress and results in better health (Adler et al., 2000; Cloutier et al., 2013; Sapolsky, 2005; Sherman et al., 2012). Not surprisingly, social psychologists have documented that the ways in which high-ranking individuals think, feel, and behave are distinct from low-ranking individuals (S. T. Fiske, 2010; Galinsky, Rucker, et al., 2015; Keltner et al., 2008; Magee & Galinsky, 2008; Piff et al., 2018; Rucker et al., 2018; Van Vugt & Smith, 2019).

However, as the above quotes illustrate, cultural contexts might differ as to how they expect high-ranking individuals to embody their role. On the one hand, at least some cultural contexts seem to emphasize the importance of looking beyond oneself and to exert influence with other people’s needs in mind. On the other hand, at least some cultural contexts seem to also emphasize the importance of looking inside oneself and to lead with a unique vision. In this review of the social and cultural psychological literature on social rank, we investigate high ranks’ psychological and behavioral tendencies toward self- and other-orientation. We propose a cultural psychological approach to social hierarchy positing that rank differences are embedded within larger cultural meaning systems. They shape how high rank is attained or conferred and also how social hierarchy affords behavior and psychology. We propose that these processes can result in culturally divergent as well as shared manifestations of social hierarchy. We will apply a cultural psychological approach to examine manifestations of social hierarchy in two specific cultural meaning systems, Western (especially American) and East Asian cultural contexts, investigating processes at the collective, interpersonal, and individual level.

Our review makes a series of significant contributions to the social hierarchy literature. First, our theoretical framework suggests that high-ranking individuals’ self-orientation does not necessarily need to be accompanied by low levels of other-orientation. Indeed, our review will demonstrate that the cognitive and behavioral tendencies of high-ranking individuals from East Asian cultural contexts, in contrast to their counterparts from Western cultural contexts, are guided by both self- and other-orientation. Thus, we improve the understanding of high rank in East Asian cultural contexts, and we also elucidate how the social hierarchy in Western cultural contexts is embedded in a unique cultural meaning system. Our framework points to potential cultural differences in the consequences of having high rank, which have important mental and physical health implications. Taken together, our review offers a stepping stone to more comprehensively study cultural variations in social hierarchy across diverse cultural contexts, and we provide a number of avenues for future research.

## Theoretical Framework

### Definition of Social Hierarchy

We define social hierarchy as a rank order of individuals along one or several socially valued dimensions (Magee & Galinsky, 2008). Hierarchies establish the social rank that an individual occupies relative to other individuals. Rank orders mean that at least one individual is subordinate to one other individual and vice versa that at least one individual is superior to one other individual. Humans consensually agree on a set of characteristics that are associated with superior rank. In other words, socially shared beliefs define those characteristics that group members defer to (Berger et al., 1972, 1998; Ridgeway, 2001; Ridgeway & Berger, 1986).

An important implication of the socially shared nature of social hierarchies is that hierarchies exist in many different forms. For example, social hierarchy can be based on tangible characteristics such as control over valued resources (i.e., social power, e.g., French & Raven, 1959; Galinsky, Rucker, et al., 2015; Keltner et al., 2003), a structural position of an individual in society (i.e., socioeconomic status, e.g., Côté, 2011; Kraus et al., 2012; Piff et al., 2018), or more intangible characteristics, such as other’s respect and admiration (i.e., sociometric status, e.g., C. Anderson et al., 2012; C. Anderson & Kilduff, 2009). Although some aspects and forms of the social hierarchy are likely to be shared across cultures, humans are a species rich in sociocultural contexts as to how best to approach and coordinate social living as well as how to sort group members into higher and lower ranks (Heine, 2012). Thus, currently missing from the social hierarchy literature is a compelling cultural lens providing some initial insight as to how social hierarchy is perceived across different societies, and what basis of social status may be valued differently in different cultural contexts.

### A Cultural Psychological Approach to Social Hierarchy

**Figure 1.**
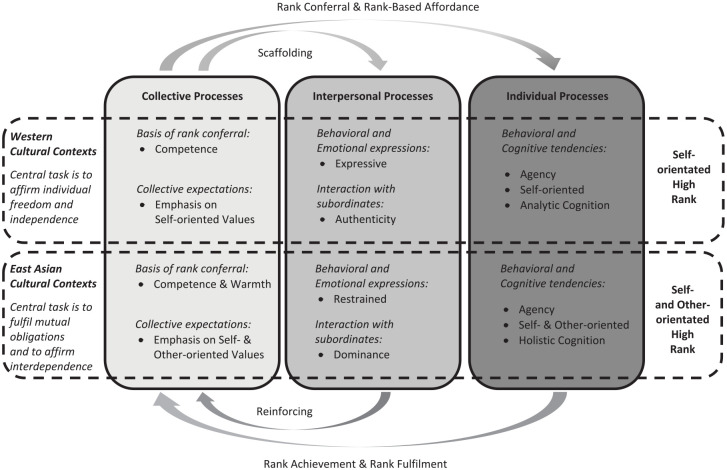
The Social Hierarchy Across Cultures Model.

While social hierarchies are ubiquitous and fundamental to social living in many cultures, a cultural psychological approach to social hierarchy suggests that social hierarchy is located within a particular cultural meaning system (Miyamoto et al., 2018; Stephens et al., 2012). Cultural contexts shape social hierarchy by symbolically defining what goals and tasks are considered prestigious and respectable (Kitayama et al., 2009; Shweder, 1991, 2003). These cultural ideals serve as the bases of rank-ordering in a given culture because they can shape individual’s beliefs as to who should occupy higher (lower) ranks and how high- (low-) ranking individuals should and can behave. Such a cultural meaning system can also shape the manifestations of social hierarchy through at least two processes: (a) a process through which those who pursue culturally valued and esteemed goals and tasks more readily attain high ranks compared to those who do not engage in such tasks (i.e., rank attainment/conferral) and (b) a process through which high-rank environments afford and expect individuals to pursue culturally valued and esteemed goals compared with low ranked environments (i.e., rank-based affordance/fulfillment). We propose that these processes can result in culturally divergent as well as shared manifestations of social hierarchy (Figure 1).

First, a cultural meaning system can shape how ranks are *attained and conferred*. Social identity theory of leadership suggests that individuals who are prototypical and normative of the group are more likely to emerge and endure as leaders (Hais et al., 1997; Hogg, 2001; Hogg et al., 2012). In addition, functional theories of social hierarchy suggest that individuals willingly confer high ranks to those members of a group who can effectively solve the specific collective tasks shared by the group (Berger et al., 1998; Van Vugt, 2006). These theories suggest that individuals who pursue the type of tasks valued and normative in the respective culture are likely to attain high ranks, whereas those who are not pursuing or unable to pursue such tasks are likely to be given lower ranks. Such a process can lead to cultural divergence in the kinds of individuals who occupy high ranks when normative tasks differ across cultures. At the same time, the same process can also lead to cross-cultural similarities in the kinds of individuals who occupy high ranks when similar tasks are valued in groups regardless of cultural contexts, such as general competence to accomplish a goal the group is facing.

Second, a cultural meaning system can also shape how social hierarchy *affords behavior and psychological processes* once individuals occupy different ranks. Social psychological research has suggested that individuals with high ranks have more access to valued resources, are freed from constraints, and thus afforded with a greater propensity toward goal-directed behavior (Galinsky et al., 2003; Guinote, 2017; Kraus et al., 2012; Rucker et al., 2018). Greater access to valued resources facilitates goal-directed behavior by prioritizing the attainment of the individual’s focal goals (Guinote, 2007b). Thus, high-ranking individuals are likely to pursue their own goals and to exert personal agency across cultures. At the same time, culture-specific goals and ideas are made chronically and temporarily accessible in a given cultural context (Oyserman & Lee, 2008). Thus, high-ranking individuals are also likely to focus on pursuing goals that are made accessible in a given cultural contexts (Guinote et al., 2012). This process should lead high-ranking more than low-ranking individuals to pursue culturally valued goals. Therefore, while the greater resources available to high-ranking individuals afford them to pursue their own goals and to exert personal agency across cultures, cultural differences in the type of goals highlighted in the given cultural context can also afford them to pursue culturally divergent goals.

Once established, the link between cultural contexts, social hierarchy, and its behavioral and psychological manifestations can mutually reinforce each other and thus operate in a self-sustaining way (Bourdieu, 1984; Stephens et al., 2012). Sociocultural practices and environments (e.g., socialization and workplace setups) of high-ranking individuals tend to reflect the goals and tasks they pursue (Bernstein, 1971; Kohn, 1969). This will instill, encourage, and mandate the corresponding kinds of goal-pursuit among individuals who participate in such practices. Furthermore, the dominant cultural meaning system and mainstream institutions (e.g., education system or workplaces) are structured to reflect the goals and task characteristics of high-ranking individuals (Stephens et al., 2014). This provides an advantage to high-ranking individuals and thus reinforces the hierarchical relationship between the higher and the lower ranks.

In sum, we propose that there are both cultural differences and similarities in behavioral and psychological manifestations of social hierarchy (Figure 1). Cultural similarities are expected for the propensity and ability to pursue goals. At the same time, cultural contexts prescribe the nature of goals and tasks considered normative, ideal, and respectable within each culture, thus leading to culturally divergent manifestations of social hierarchy. In the subsequent sections, we first define the psychological constructs that are central to the understanding of psychological manifestations of social hierarchy, and we will then apply a cultural psychological approach to examine manifestations of hierarchy in two specific cultural meaning systems: Western (especially American) and East Asian cultural contexts.

### Self-Orientation and Other-Orientation

One of the fundamental distinctions made by many researchers to understand the relationships between social hierarchy and psychological processes is the orientation toward oneself versus others (S. Chen, 2020; see Kraus et al., 2012 for individualistic versus external orientations; see Rucker et al., 2018 for agency and communality; see Winter, 1973 for personalized and socialized power motives). Psychological processes and behaviors that support and promote one’s own person constitute the core of orientations toward the self (i.e., self-orientation). For example, the pursuit of self-set goals and self-esteem promote the self and thus are examples of self-orientation. On the other hand, psychological processes and behaviors that support and promote others, the relationships with others, and the group comprise orientations toward others (i.e., other-orientation). Examples include helping others, adjusting to others, and fulfilling one’s responsibility to others, as well as enforcing rules or norms and concerns for face, which likely promote or maintain one’s relationships with others or promote others’ and group goals.

While self-orientation and other-orientation are often viewed as opposites (e.g., Rucker et al., 2018), it is important to note that they are conceptually distinct and may coexist with each other. For example, when self-orientation and other-orientation require a trade-off (e.g., one needs to sacrifice one’s interest to help others or one pursues self-interest that harms others), self-orientation and other-orientation are mutually exclusive (Volz et al., 2017). However, people can help others to fulfill their self-interests (Cialdini et al., 1987) or can act assertively to negotiate for others (Amanatullah & Morris, 2010). In such situations, self-orientation and other-orientation can coexist.

### Manifestations of Social Hierarchy in Western and East Asian Cultural Contexts

We follow Kroeber and Kluckhohn (1952) and Adams and Markus (2004) in defining culture as a historically derived system of explicit and implicit patterns of ideas and their socially shared embodiment in artifacts, practices, and institutions, which are produced by behavior and which also shape behavior. A core premise of this definition is that culture is a dynamic system that involves mutually sustaining relationships between cultural patterns and individual psychological activity (Shweder, 1991). Thus, it is important to note that differences in behavioral and psychological processes observed across cultures are assumed to reflect differences in cultural patterns that afforded such individual responses. As such, culture should not be mistaken as fixed essences of individuals, reified social categories, or stereotypical descriptions of groups (Adams & Markus, 2004). Instead, cultural meaning systems are characterized by ideas that have been shaped by ecological and historical factors, have been accumulated and changed over time, and manifest both in individual minds and in cultural products, practices, and institutions (A. P. Fiske et al., 1998). From this perspective, cultural dimensions, such as comparisons between Western and East Asian cultures, constitute rough approximations of these dynamic realities and diverse social representations.^1^

For example, Western cultural contexts, such as the United States, are characterized by the emphasis on individual freedom and the notion of the self that is defined by unique internal attributes separated from the surrounding contexts (de Tocqueville, 2004; Markus & Kitayama, 1991; Triandis, 1995). In contrast, East Asian cultural contexts with a Confucian influence are characterized by the emphasis on mutual obligations within hierarchical relationships and the notion of the self that is inherently connected to and embedded in social relationships (Hofstede & Bond, 1988; Markus & Kitayama, 1991; Triandis, 1995). Western and East Asian cultural meaning systems thus differ in the tasks and goals that are considered ideal, prestigious, and respectable (Kitayama et al., 2009; Shweder, 2003). In Western cultural contexts, promoting and realizing the uniqueness of the self and self-set goals (i.e., self-orientation), which affirm individual freedom and independence, are tasks that are valued and sanctioned. In East Asian, Confucian cultural contexts, however, promoting and maintaining the relations between the self and the others (i.e., other-orientation), which fulfill mutual obligations and affirm interdependence, are tasks that are valued and sanctioned.^2^

Cross-cultural studies have documented variations between Western and East Asian cultural contexts in a wide range of behavioral and psychological processes that reflect such culturally sanctioned tasks. For example, European Americans are more likely than East Asians to take an insider perspective on themselves (D. Cohen et al., 2007), express themselves (Kim, 2002; Kim & Sherman, 2007) and their emotions (Matsumoto et al., 2008), exert influence on the environment (Morling et al., 2002), and show analytic attention and thinking styles (Nisbett et al., 2001). On the contrary, East Asians are more likely than European Americans to adjust themselves to take an outsider perspective on themselves (D. Cohen et al., 2007), suppress their emotions (Matsumoto et al., 2008), adjust to their environment (Morling et al., 2002), and show holistic attention and contextual thinking styles (Nisbett et al., 2001). Notably, most of the studies showing cultural differences in behavioral and psychological processes are based on college students; thus, it is not clear how individuals who belong to different positions in the social hierarchy within each culture would think and behave.

A cultural psychological approach to social hierarchy offers two propositions about how social hierarchy manifests in relation to such behavioral and psychological processes that reflect culturally sanctioned goals. First, as discussed in the previous section, high-ranking individuals across cultures should have the propensity and ability to pursue their own goals and to exert personal agency due to resources associated with their high ranks (Galinsky et al., 2003; Guinote, 2007b; Kraus et al., 2012; Rucker et al., 2018; Van Vugt, 2006). Thus, we propose that those with high ranks are likely to engage in self-oriented behavior and psychological processes across Western and East Asian cultural contexts. At the same time, culture can make it more likely for high-ranking individuals to pursue goals and tasks valued within the given cultural context by conferring individuals who pursue such goals with high rank and also by making such goals salient and accessible. Thus, we propose that high-ranking individuals in Western cultural contexts should be especially likely to engage in self-oriented behavior and psychological processes. In contrast, high-ranking individuals in East Asian cultural contexts should be especially likely to engage in other-oriented behavior and psychological processes. The co-existence of other- and self-oriented goals among high ranks in East Asian cultural contexts is in fact in line with the Confucian teaching, which emphasizes both diligence/achievement (Bond & Hwang, 1986) and responsibilities/obligations associated with one’s roles (Fu et al., 2008).

One of the implications of a cultural approach to social hierarchy is that even within Western (or East Asian) cultural contexts, culture is not uniform. Rather, multiple cultural contexts exist (e.g., socioeconomic status, region, religion; A. B. Cohen, 2009) within the broader culture. Individuals are embedded in and constantly interacting with multiple cultural contexts, which can lead to individual variations in behavioral and psychological processes. For example, although both high-ranking individuals and low-ranking individuals in East Asian cultural contexts are exposed to the central values of interdependence highlighted in East Asian cultures, how these cultural ideas are embodied and manifest in proximal cultural contexts can vary depending on their position in the social hierarchy. Such variations in specific cultural contexts can result in differences in the behavioral and psychological processes of high-ranking and low-ranking individuals, which in turn can work to reinforce social hierarchies.

In this review, we comprehensively analyze evidence on cultural differences and similarities in manifestations of social hierarchy by focusing on three domains: collective, interpersonal, and individual processes of high-ranking individuals (Table 1). At the level of collective processes, we propose that there are cultural similarities and differences in the expectations for individuals who are conferred with and attain high ranks. High-ranking individuals in Western cultural contexts are expected to be competent and influential. Although high-ranking individuals in East Asian cultural contexts are expected to be competent to a certain degree, they are also expected to be warm and responsible. At the level of interpersonal processes, we argue that the nature of interpersonal interactions of high-ranking individuals depends on culture. Interpersonal interactions of high-ranking individuals in Western cultural contexts are expressive and disinhibited, whereas those in East Asian cultural contexts are restrained and dominant. Finally, at the level of individual processes, we suggest that there are both cultural similarities and differences in psychological processes of high-ranking individuals. High-ranking individuals in Western cultural contexts tend to be self-oriented and analytic, whereas high-ranking individuals in East Asian cultural contexts tend to be both self-oriented and other-oriented / holistic. In the subsequent sections, we review the relevant evidence in each of these domains.

**Table 1.** Collective, Interpersonal, and Individual Processes of High-Ranking Individuals in Western and East Asian Cultural Contexts.

	***Self-orientation in Western cultural contexts***	***Self- and other-orientation in East Asian cultural contexts***
**Collective Processes**		
*Rank conferral*	Competence Norm violator	Competence & Warmth Norm abiding
*Collective representations and expectations*	Influence/ Entitlement Leading with one’s own vision Trailblazing Self-oriented values	Responsibility Leading with authority & benevolence Trailing-behind Self- & other-oriented values
**Interpersonal Processes**		
*Behavioral/emotional expressions*	Expressive High arousal positive Less anger expression	Restrained Low arousal positive More anger expression
*Interactions with subordinates*	Authenticity	Dominance (punishment)
**Individual Processes**		
*Psychological processes*	Agency Self-oriented Analytic cognition	Agency Self- & other-oriented Holistic cognition

## Review of Findings on Social Hierarchy Across Cultures

### Collective Processes—Who Gets to Attain High Rank and How Should They Behave?

In this section of our review, we will assess cultural similarities and differences in the expectations for high-ranking individuals and for those who are conferred with and attain high ranks.

#### Rank Attainment/Conferral

One way in which culture influences social hierarchies is through collective processes that govern rank conferral. Individuals are conferred higher social rank to the extent that others agree that they possess characteristics and perform tasks that are valued in the group. Leadership positions, for example, are based on the qualities that are expected to be the most beneficial for the success of a given group (Berger et al., 1972; Emerson, 1962). In other words, groups have an understanding as to what characteristics and tasks are valued, and they assign individuals to high ranks the more they embody these characteristics. Unsurprisingly, more prototypical group members are more likely to achieve leadership positions because prototypical characteristics are likely to be normative and valued by group members (Hais et al., 1997). A person’s social rank is therefore not so much a tangible characteristic possessed by the actor but rather the collective evaluation in the eyes of observers.

In the American cultural context, for example, the dominant ideology is the belief in meritocracy (Kluegel & Smith, 1986; Lamont, 1999), which legitimizes superior rank for individuals with outstanding performance. One key characteristic that may signal merit is a person’s competence. Indeed, when individuals compete for high social ranks, those candidates who are perceived as more competent are more likely to win the competitions and even to be elected into political office (Todorov et al., 2005). For example, in one series of studies, Ballew and Todorov (2007) presented participants with the faces of two unfamiliar political candidates who opposed each other in American elections and asked participants to pick the one who looked more competent. Participants’ judgment of the competence predicted actual election outcomes more than two out of three times. Indeed, facial competence seems to be a highly robust and specific predictor of achieving political leadership in the United States (Olivola & Todorov, 2010). In fact, the perceived competence of a Western leadership candidate does predict not only election outcomes but also company profits (Rule & Ambady, 2011).

Yet, to the extent that shared beliefs about valuable characteristics and tasks vary across cultures, social rank can be based on different dimensions. It is possible that perceived competence is especially likely to matter in individualistic cultures where unique individual performance is especially valued. In fact, although perceived competence of faces predicted voting outcomes in hypothetical elections in both the United States and South Korea when no other information was available, perceived competence was a better predictor of electoral outcomes in actual elections in the United States than in South Korea (Na et al., 2015). Furthermore, whereas perceived facial competence predicted company profits of U.S. companies (Rule & Ambady, 2008), this was not the case for Japanese companies (Rule et al., 2011). These results suggest that although perceived competence is relevant to rank conferral to some extent across cultures, it is likely weighted against other characteristics valued in other cultures. For example, research has demonstrated that perceived warmth might be crucial for the attainment of high social ranks in East Asia (Misumi & Peterson, 1985; P. B. Smith et al., 1989; Yamaguchi, 1994), and the perceived warmth of Japanese candidates predicted how likely they were to be elected in actual elections (Rule et al., 2010). Therefore, perceived warmth seems to play an important role in rank conferral in East Asian cultural contexts, possibly because maintaining relationships among the group is a task valued in collectivistic cultures.

This interpretation resonates with another recent finding. Stamkou and colleagues (2019) surveyed more than 2,300 participants across 19 countries, asking them whether norm-abiding or norm-violating individuals are perceived to have higher social rank. One way to think about norm-abiding versus norm-violating individuals is that the former are blending into their group preserving the group’s social cohesion, whereas the latter are sticking out of the group portraying their individuality. Based on this conceptualization, it seems reasonable to predict that norm-abiding individuals might be perceived as higher in social rank in more collectivistic cultures because their behavior is more aligned with the cultural goal of interdependence. In contrast, it seems reasonable to predict that norm-violating individuals might be perceived as higher in social rank in more individualistic cultures because their behavior is more aligned with the cultural goal of independence. Indeed, the results supported these predictions. Norm-abiding individuals were perceived as more powerful in more collectivistic cultures, whereas norm-violating leaders were perceived as more powerful in more individualistic cultures (Stamkou et al., 2019). Thus, what characteristics predict high social rank varies significantly across cultures. As we shall see next, these cultural similarities and differences about who is to attain high social rank also influence expectations for how high-ranking individuals behave.

#### Representations and Expectations for High-Ranking Individuals

A significant body of research in cross-cultural psychology demonstrates that the content of norms differs across cultures. In other words, individuals in different cultures believe that individuals should behave differently. The studies reviewed in the previous section showed cultural similarities and differences in who is likely to be conferred with high social ranks. At the same time, cultural contexts can also influence how individuals who are (already) in a position of high rank are perceived and expected to behave. For example, research hints at important cultural differences between North American and East Asian cultural contexts in their conceptualizations of power. While the mental representation of power focuses on influence and entitlement in Western cultural contexts, it is associated with responsibility in East Asian cultural context, leading individuals to have different expectations for high-ranking individuals. For example, using subliminal priming procedure, Zhong and colleagues (2006) assessed participants’ automatic association of power with either entitlement-related words, such as merit or deserve, or responsibility-related words, such as dependable or duty. In this study, participants were asked to respond as quickly and as accurately as possible to decide whether a letter string on screen was either a word or a non-word. Crucially, immediately before the letter strings appeared on screen, the researchers flashed the word “power” or the control word “paper” for a very short duration (86 ms) and outside of participants’ awareness on screen. Results showed that following subliminal priming with “power,” American participants were faster to respond to entitlement-related rather than responsibility-related words, whereas East Asian participants were quicker to respond to responsibility-related rather than entitlement-related words (Zhong et al., 2006). These findings suggest that the American cultural context fosters a stronger semantic association of power with entitlement-related words, and therefore participants were able to respond quicker to these. That is, to American participants, power means to be self-focused. In contrast, East Asian cultural contexts seem to foster a stronger semantic association of power with responsibility-related words, and thus East Asian participants were able to respond quicker to those. Thus, to East Asian participants, power means to be other-focused. Similarly, other research shows that while in more (vertical) individualistic cultures, power is conceptualized in terms of personalized power (i.e., power for advancing one’s personal position), in more (horizontal) collectivistic cultures, power is conceptualized in terms of socialized power (i.e., power to benefit others; Torelli & Shavitt, 2010).

Such cultural differences in mental representations of what it means to be powerful and more generally to be higher in social rank can influence Americans’ and East Asians’ expectations of high-ranking individuals, which are most apparent when studying leadership in organizational settings. For example, one crucial cultural difference between East Asian and Western cultural contexts centers around the concept of paternalistic leadership (Dorfman et al., 1997). Farh and Cheng (2000) define paternalistic leadership as “a style that combines strong discipline and authority with fatherly benevolence” (p. 91). Paternalistic expectations in East Asian cultural contexts emphasize the responsibility that leaders have for their subordinates (Pellegrini & Scandura, 2008). The care, support, and protection of the paternalistic leader fit the East Asian cultural value of interdependence. Thus, Japanese employees view paternalistic leadership behavior positively (Uhl-Bien et al., 1990), and especially the benevolent aspects of paternalistic leadership which are expressed through a holistic concern for the subordinates’ personal and family well-being (Farh & Cheng, 2000). For example, P. B. Smith and colleagues (1989) found that Japanese leaders were expected to attend to the personal needs of their subordinates and socialize with them after working hours.

The type of behaviors and attributes perceived to be important for leaders have been examined across cultures. Data from middle managers across 62 cultures, collected in the GLOBE Project (House et al., 2004), showed that while charismatic attributes that inspire and expect high-performance outcomes from the subordinates were rated to be the most desirable dimensions for an outstanding leader across cultures, team-oriented attributes that emphasize building relationships among team members were perceived to be as desirable as charismatic attributes in Confucian Asian and Latin American cultural contexts (Javidan et al., 2006). For example, while both American and Chinese middle-managers valued leaders oriented toward excellence and performance improvement, Chinese managers also valued leaders who were fraternal with their subordinates (Javidan et al., 2006). Such findings are also in line with the Performance-Maintenance Theory of leadership (Misumi & Peterson, 1985), which suggests that effective leaders in the Japanese cultural context are perceived to be high on both performance function, which focuses on reaching group goals, and maintenance function, which focuses on maintaining the social stability of the group.

The cultural differences in the expectations for leaders are also reflected in the amount of responsibility leaders are expected to take when things go wrong in the organization. For example, in Japan compared to the United States, managers are more likely to be blamed for organizational failures, even when they are not directly involved in the failures (Zemba et al., 2006). In fact, Japanese leaders have internalized cultural expectations about them assuming responsibility to the degree that they judge themselves as a key factor when things go wrong. For example, in one study, when asked to imagine that they were the chief executive officer with the task to fire employees due to economic pressures, Japanese were more likely than Americans to report feeling responsible for the fired employee as well as for their families (Maddux & Yuki, 2006). High-ranking people in East Asian cultural contexts seem to feel more responsibility for the consequences of their decisions on other individuals than high-ranking people in Western cultural contexts.

To best assume responsibility, cultures may also vary in where they represent their leaders to stand. One study tested this possibility empirically. In this study, participants saw a visual representation of a group of individuals, and they had to indicate the position of the leader (Menon et al., 2010). While East Asian participants from Singapore were more likely to select the individual in the rear (standing behind the group) as the leader, Western participants from the United States were more likely to select the individual in front of the group as the leader. This suggests that mentally representing a leader in front of the group might express how leaders in Western cultural contexts are expected to pursue opportunities, assert their own will, take risks, and innovate. In contrast, mentally representing a leader as standing behind the group might express the expectation of leaders to focus on the group, the relational goals, protecting the group from threat, and maintaining social harmony. Thus, it seems that not only do expected leadership characteristics vary across cultures but also their mental representation of where leaders stand. As we shall see next, these expectations are internalized early on through socialization practices.

#### Socialization of Expectations Associated With Different Ranks

One pathway through which the cultural differences in the expectations for high-ranking individuals are instilled in individuals may be via socialization practices. Socialization practices through which high- (versus low-) ranking individuals raise their children tend to convey certain expectations for their children (Bernstein, 1971; Kohn, 1969). Studies have suggested that some expectations associated with varying social ranks differ across cultures, whereas others are shared across cultures.

Qualitative studies done in the United States and Japan have illustrated cultural differences in expectations conveyed through socialization practices of high- versus low-ranking individuals. Ethnographical studies in the United States suggested that the socialization practices of high-ranking individuals tend to emphasize self-confidence and uniqueness compared with the socialization practices of low-ranking individuals (Kusserow, 1999, 2012). In contrast, ethnographical studies in Japan suggested that educational systems characteristic of high-ranking individuals (e.g., elite college, high school for college-bound students) tend to highlight group cohesion and the associated responsibilities than educational systems characteristic of low-ranking individuals (e.g., low-tier college, high school where most of its graduates enter the workforce) (Borovoy, 2010; Slater, 2010).

A similar pattern was found in quantitative studies. Studies done in the United States showed that higher social class parents tend to value the self-direction (e.g., happiness and curiosity) of their child, whereas lower social class parents tend to value conformity (Kohn, 1969). On the contrary, according to the perception of students attending various high schools in Japan, higher social class parents were more likely than lower social class parents to highlight socially oriented values, such as conformity to social standards and adherence to socially expected roles (Kataoka, 1987). At the same time, in the same study, Japanese parents with the higher social class were also perceived to emphasize personal values, such as personal achievement. Thus, while high-ranking parents in the United States mainly value self-orientation of their children, high-ranking parents in Japan seem to *also* value other-orientation of their children.

A direct cross-cultural comparison of socialization values across 60 nations found cultural similarities and differences in socialization values associated with varying socioeconomic status (Miyamoto et al., 2018). Respondents across 60 nations were asked to pick up to five values that they considered especially important for children to learn at home. Some values were self-oriented (e.g., self-expression, hard work), while others were other-oriented (e.g., the feeling of responsibility, unselfishness). Across 60 nations, in general, there was a positive association between socioeconomic status (SES; i.e., educational attainment) and the endorsement of self-oriented socialization values. At the same time, there was also a positive association between SES and the endorsement of other-oriented socialization values in Confucian cultural contexts, such as Japan, China, and Korea, whereas the association was negative in Frontier cultural contexts, such as the United States and Australia. These findings suggest that while socialization practices of high-ranking individuals in the United States mainly involve the emphasis on self-oriented values, socialization practices of high-ranking individuals in East Asian cultural contexts also include the emphasis on other-oriented values.

#### Summary—Collective Processes

In this section, we reviewed evidence speaking to the idea that one way in which culture influences social hierarchy is through collective processes. While in Western cultural contexts, individuals hold beliefs that the most competent individual will achieve high social ranks, the findings showed that in East Asian cultural contexts, such belief is complemented by the importance of other-orientation (i.e., warmth) for attaining high social ranks. Similarly, once in a position of high rank, culture yields a strong influence over what is expected of the high-ranking individual. Here again, our review yielded marked differences in self- and other-orientation. While entitlement was an automatically activated concept of high rank in Western cultural contexts, in East Asian cultural contexts, high rank meant first and foremost responsibility. Cultural differences in socialization practices, we showed further, might be one pathway through which these different expectations are instilled in individuals. Next, we turn our attention to how cultures’ influence on rank conferral and behavioral expectations will be manifest in interpersonal interactions.

### Interpersonal Processes—How Is High Rank Signaled and Expressed?

In this section of our review, we will assess how culture influences manifestations of high rank when high- and low-ranking individuals interact.

#### Behavioral Expressions Versus Behavioral Restraint

As seen above, cultural contexts differ in how they expect high-ranking individuals to act and to behave in social contexts. These expectations, we propose, will indeed influence behavioral manifestations of high rank across cultures. Studies done in Western/American cultural contexts have shown that high-ranking individuals tend to be more disinhibited in their behavioral expressions (Keltner et al., 2003), such as openly expressing their attitudes (C. Anderson & Berdahl, 2002) and having open body posture (Hall et al., 2005). On the contrary, it is possible that high-ranking individuals in East Asian cultural contexts may be more restrained in their behavioral expressions due to the cultural value to fit in. In fact, whereas expansive body postures (as opposed to a constricted posture) are linked to a higher sense of power among people in the American cultural context, such a link was not found among people in East Asian cultural contexts (L. E. Park et al., 2013). As seen earlier, norm-abiding individuals tend to be perceived as more powerful in more collectivistic cultures than in less collectivistic cultures (Stamkou et al., 2019). Interestingly, the scenario the authors used to describe the norm-abiding employee depicted the employee with behavioral restraint and other-orientation. Specifically, the employee arrived on time to take part in a meeting, waited to get his cup of coffee so as to not disturb his colleagues, and only spoke after his colleagues had had a chance to express themselves. Thus, in collectivistic cultures, behavioral restraint and modifying one’s behavior to accommodate others’ needs are perceived as being higher in social rank.

Assessing actual behavior, Ito and colleagues (2018) investigated the link between nonverbal restraint and leadership perceptions in Japanese University clubs. These authors video-recorded Japanese University club leaders and members talking into the camera. The video-clips were then coded for nonverbal cues of behavioral restraint (e.g., still body posture with hands behind the back) or expression (e.g., forceful gesturing). Results showed that the level of nonverbal-behavior differed between leaders and members as a function of the overarching purpose of the university club. When there was a shared understanding that the university club was task-oriented and thus required a leader to facilitate intragroup coordination (e.g., a sports club), its leader exhibited more non-verbal restraint. Furthermore, Ito and colleagues presented short extracts of the video recordings to a new set of university students and asked them to rate the leadership worthiness of the targets in the videos. Results showed that targets who displayed more nonverbal restraint but not more nonverbal expression, were perceived as worthier leaders (Ito et al., 2018). Together, these results (Ito et al., 2018; Stamkou et al., 2019) suggest that a major cultural difference between East Asian and Western cultural contexts might exist in how leaders’ nonverbal behavioral restraint is linked to higher social ranks.

#### Emotional Expressions

Expressing how one feels can be a powerful communicative tool. But cultures may differ in how they expect their high-ranking members to communicate their affective states in interpersonal contexts. While Japanese are less likely than Americans to agree that having power means that one can display any emotion one wants (Mondillon et al., 2005), the association between high rank and emotional expressions is likely to also depend on the type of emotions being expressed.

While high-ranking individuals may express positive emotions more than low-ranking individuals across cultures (Bjornsdottir & Rule, 2017), cultural contexts may shape the type of positive emotions considered to be ideal. Tsai and colleagues (2006) showed that high-arousal positive affect (e.g., excitement) is valued more in European American cultural contexts, whereas low-arousal positive affect (e.g., calmness) is valued more in East Asian cultural contexts. Expressing affective states that fit the cultural ideal has been linked to acquiring resources. For example, in a hypothetical recruitment scenario, job applicants expressing high-arousal positive affect were more likely to be hired in the United States than in China (Bencharit et al., 2019). One might thus expect that communicating affective states that are considered ideal in their own culture is linked to a higher rank. This possibility was tested by Tsai and colleagues (2016) who investigated whether leaders’ smiles reflected cultural differences in ideal affect. For example, in one study, these researchers collected photos from the official websites of American and Chinese leaders in government, businesses, and academia and coded their facial expressions. Results showed that American leaders showed more excited smiles (revealing their teeth) than Chinese leaders. Moreover, in a subsequent study, these authors were able to show that the more a national culture valued high-arousal positive affect, the more likely their leaders were to express their emotional states by displaying excited smiles (Tsai et al., 2016). These studies seem to suggest that the type of emotions valued in the given culture is more likely to be expressed by high-ranking individuals in the culture.

At the same time, high-ranking individuals also tend to express certain emotions that are not necessarily valued in their culture, such as anger in East Asian cultural contexts. Anger can signal a person’s frustration when personal goals are blocked and help to re-establish one’s self-efficacy, or it can be a warning signal to low-ranking conspecifics affirming higher social rank. J. Park et al., (2013) investigated self-reported anger expressions among large-scale representative samples of Japanese and American adults. In line with the independent notion of the self, defined by its own attributes, in Western cultural contexts, these researchers found that lower status individuals from the United States were more likely to report anger expressions in their daily lives, which was partly explained by larger frustrations they felt. In contrast, in East Asian cultural contexts, where social harmony with others and interpersonal belongingness are highly valued, anger expressions might be perceived as disruptive. In such a cultural context, only those who occupy high ranks may express anger, as part of their role is to maintain the socio-hierarchical structure of their groups. Indeed, J. Park et al., (2013) found that anger expressions were more frequently reported by high-ranking individuals in Japan, and this relationship was mediated by their perceived decision-making authority.

#### Interpersonal Interactions Between High- and Low-Ranking Individuals

The role of high-ranking individuals in East Asian cultural contexts to express anger may also be reflected in how they tend to interact with their subordinates. In fact, survey data suggests that the Japanese were more likely than Americans to think that it is appropriate to express behavioral dominance when interacting with low-ranking individuals (Matsumoto, 2007). While in the American cultural context, high-ranking individuals tend to freely express their authentic self (Kraus et al., 2011), high-ranking individuals in East Asian cultural contexts might be responding to the situational demands to ensure cohesion and interdependence in their groups, which may sometimes require enforcement of rules. In support of this idea, in specific circumstances, East Asian leaders are expected to display their power by setting rules and even issuing punishment (Aycan, 2006; Farh et al., 2006).

For example, Kuwabara and colleagues (2016) studied the extent to which high-ranking individuals punish group members in China and the United States. They asked participants to complete a leadership questionnaire. Ostensibly based on their leadership score, in the high-rank condition, one participant was chosen to be the powerful punisher, a position that commanded a fair amount of respect from workers. In the same rank condition, however, one participant was chosen at random to be the punisher, and this person was told that they had the same rank as their workers. The authors found that participants from China and India assigned to the high rank compared with the same rank condition were more likely to punish group members than their American counterparts. In this particular study, the punishment was part of a public goods game, so it is possible and perhaps likely that punishment served a social function to ensure group coherence and order through punishing free-riding and deviant behaviors (see also C. Chen et al., 2021).

Initial support is lent to this idea by a recent study that investigated levels of social assurance within Western and East Asian work environments (Ito et al., 2023). In one study, working adults from Japan and the United States were asked to think about either a dominant or a prestigious leader at work. Japanese but not American working adults felt greater social assurance when imagining working under a dominant leader. This was the case because they perceived the dominant leader to have greater power to punish free-riding in their workplace. Thus, this study provides causal evidence that work environments with dominant leaders increased perceptions of social assurance in Japan but not the United States (Ito et al., 2023). Moreover, in the study reviewed earlier, Menon and colleagues (2010) primed a mono-cultural sample of American MBA students with concepts of opportunity versus threat. They found that participants made to think about the key opportunities of their company increased the frequency of selecting the individual standing in front as the leader, whereas participants made to think about the key threats to their company increased the frequency of selecting the individual standing in the rear as the leader (Menon et al., 2010). Taken together, then these results provide initial empirical support for the idea that in environments characterized by strong in-group vigilance, as is the case in East Asian cultural contexts (Liu et al., 2019), a dominant leader may serve the important function of ensuring group cohesion.

The cultural appropriateness of being a benevolent and punishing leader in East Asian cultural contexts is further illustrated by the organizational literature on paternalism. Indeed, paternalism has different components, including benevolent, moral, and authoritarian dimensions (Farh & Cheng, 2000; Pellegrini & Scandura, 2008). While paternalistic leadership is associated with employees’ job satisfaction in collectivistic cultures, such as India, it is unrelated to employees’ job satisfaction in individualistic cultures, such as the United States (Cheng et al., 2014). Moreover, while it is generally believed that abusive supervision behavior leads to reduced well-being among subordinates, this is not the case in China (Lin et al., 2013). This was because employees in China had a different construal of their leaders, emphasizing the power differential between them. Thus, consistent with the empirical evidence on higher rank and anger expressions in Japan (J. Park et al., 2013), leaders in collectivistic cultures might not always restraint their actions and behaviors, but rather adapt them to their social role and the situational demands. If the situation demands that they will provide directions, coordination, and perhaps even express their anger, they will do so.

#### Summary—Interpersonal Processes

In this section, we reviewed evidence speaking to the idea that another important way in which culture influences social hierarchy is through interpersonal processes. First, we reviewed evidence that while in Western cultural contexts, high social rank is associated with behavioral and emotional expressions, in East Asian cultural contexts, high social rank is associated with behavioral and emotional restraint. Interestingly, the literature also shows that anger expressions are more frequent among high-ranking people in East Asian cultural contexts. Our review suggests that one possibility therefore may be that anger expressions and punishment serve a social function to ensure group cohesion and order through punishing free-riding and deviant behaviors. Thus, high-ranking individuals in collectivistic East Asian cultural contexts might not always restraint their actions and behaviors but rather adapt them to their social role and the other-oriented situational demands. Given the important cultural differences in collective expectations and interpersonal behavioral tendencies, one would also expect that culture influences the psychological tendencies of high-ranking individuals. Next, we turn our attention to how cultures influence the psychological consequences of high rank.

### Individual Processes—What Are the Psychological Consequences of High Rank?

Through both collective processes (e.g., rank attainment/conferral, socialization practices, interpersonal expectations) and interpersonal processes (e.g., rank signaling, behavioral and emotional expressions), cultural contexts can shape how social hierarchy manifests in psychological processes of individuals who belong to different positions in the social hierarchy. In particular, cross-cultural studies have examined psychological correlates and consequences of social hierarchy in relation to self- and other-oriented psychological processes and analytic and holistic cognition.

#### Psychological Self- and Other-Orientation

Other-orientation, the psychological tendency to put oneself in others’ shoes and to feel what they feel, is one crucial skill for those in high-ranking positions to navigate interpersonal demands. For example, greater psychological other-orientation is associated with reduced bias in thought processes (Galinsky & Moskowitz, 2000; Todd et al., 2011), improved social bonds (Stephan & Finlay, 1999), interpersonal coordination (Galinsky et al., 2005), better decision-making through increased information-sharing (Galinsky, Todd, et al., 2015), better negotiation outcomes (Galinsky & Mussweiler, 2001), and increased motivation, satisfaction, and well-being at work (J. Choi, 2006; Scott et al., 2010). In short, other-orientation can be a pivotal psychological tendency for the success or failure of those who have power. Yet, a cultural psychological approach to social hierarchy proposes that the extent to which high-ranking individuals engage in other-orientation may differ across cultures. On the one hand, individualistic cultural contexts, such as the United States, may downplay the importance of psychological other-orientation in high-ranking individuals, as they promote goals of uniqueness, competition, and the pursuit of individual goals. On the other hand, collectivistic cultural contexts, such as Japan, may emphasize the importance of psychological other-orientation for efficient high-ranking individuals, as they promote interpersonal connectedness and the pursuit of goals to the group’s benefit.

Indeed, past research on high-ranking individuals has shown increased self-orientation and their reduced other-orientation (Kohn, 1969; Kraus et al., 2012; P. K. Smith & Bargh, 2008; Stephens et al., 2014). These studies have shown that high-ranking individuals are more likely to have high self-confidence (Kohn, 1969), take actions to pursue own goals (Galinsky et al., 2003), seek unique choices (Stephens et al., 2007), and have a focused, analytic thinking style (Na & Kitayama, 2011). At the same time, high-ranking individuals were found to be *less* other-oriented, as indicated by lower conformity (Kohn, 1969), lower perspective-taking (Blader et al., 2016; Galinsky et al., 2006), being less likely to adjust themselves to others or to contexts (Galinsky et al., 2008; Kraus et al., 2011), and being less likely to listen to the opinion of others (Tost et al., 2012). Parallel patterns have been found for SES (Kraus et al., 2012; Stephens et al., 2014). Individuals with higher SES backgrounds tend to show increased self-orientation, such as higher self-confidence and higher sense of control (Lachman & Weaver, 1998). In addition, higher SES individuals also tend to show reduced other-orientation; they are less likely to engage in prosocial behavior (Piff et al., 2010) and tend to conform less to other individuals (Stephens et al., 2007).

It is noteworthy that most of the past studies that showed the link between high rank and lower other-orientation have been conducted in Western, mostly American cultural contexts, where the pursuit of self-set goals (i.e., self-orientation) is the primary cultural task. In contrast, a cultural psychological approach to social hierarchy predicts that in East Asian cultural contexts, high-ranking individuals may exhibit other-oriented psychological processes in addition to self-oriented psychological processes. On the one hand, studies that examined self-orientation in East Asian cultural contexts have found a link between higher SES and self-orientation (Hamamura et al., 2013; Ishii et al., 2017; Takemura et al., 2016). For example, a survey conducted across all the provinces in China found that higher income and higher education were associated with more value placed on influencing others (Takemura et al., 2016). In addition, across both the United States and Japan, individuals with higher subjective social status were less likely to discount future rewards relative to immediate rewards, presumably due to the resources and control they had (Ishii et al., 2017).

At the same time, studies that examined both self- and other-orientation in East Asian cultural contexts found both cultural similarities and differences. A survey of a probability sample of employed males in Japan found that individuals who occupy higher organizational ranks not only showed higher self-confidence (i.e., higher self-orientation), but also tended to conform to others’ ideas more (i.e., higher other-orientation) compared with those who occupy lower ranks (Naoi & Schooler, 1985). Analyses of large-scale international surveys based on representative samples from the United States and Japan provided a direct cross-cultural comparison of the links between SES and self- and other-orientation. While higher SES, measured in terms of both subjective social status and educational attainment, was associated with higher other-orientation, such as sympathy and support provided to others, in Japan, such associations were weaker in the United States (Miyamoto et al., 2018). At the same time, in the same surveys, higher SES was associated with higher self-orientation, such as self-esteem and goal-striving, similarly across cultural contexts. Such findings suggest that while higher SES is associated mainly with self-orientation in the United States, higher SES is associated with both other-orientation and self-orientation in Japan. Cultural differences in the link between SES and other-orientation were also shown with behavioral measures of other-orientation. When presented with others’ choices that conflicted with one’s preference, higher SES Americans were less likely to adjust to others’ choice compared with their lower SES counterpart, whereas Asians with higher SES were equally likely to adjust to others’ choice as their lower SES counterpart (Na et al., 2016). Moreover, in the same study, priming Americans to think in an interdependent way made higher SES participants adjust to others’ choice as much as their lower SES counterpart, thus suggesting the role of interdependence in the link between SES and other-orientation. Corroborating the findings above, E. Choi and colleagues (2023) recently investigated the other-orientated tendencies of powerholders across cultures. Using both archival and survey data, they found that in more collectivistic cultural contexts powerholders reported greater other-orientation than in more individualistic cultural contexts. Thus, it seems that cultural contexts substantially influence both the self- and other-oriented psychological tendencies of higher and lower ranked individuals.

#### Analytic and Holistic Cognition

Divergence in the psychological orientations of high-ranking individuals across cultures has implications for cognitive processes, too. Processing focal information without paying too much attention to contexts (i.e., analytic cognition) may help a person to pursue their own goals without being distracted by peripheral or contextual information (Guinote, 2007a). In contrast, processing contextual information and paying attention to the relationship between the focal objects and the field (i.e., holistic cognition) may help a person to attend to and fit into social contexts. In fact, previous studies have shown that individuals who attend to contextual information are more likely to show other-orientation, such as other-oriented forms of social anxiety (i.e., taijin kyofusho; Norasakkunkit et al., 2012) and higher behavioral alignment with another person (Van Baaren et al., 2004). Given these findings, one may expect that high-ranking individuals in Western cultural contexts show more analytic cognition, which serves self-orientation, whereas high-ranking individuals in East Asian cultural contexts show more holistic cognition, serving other-orientation.

Consistent with this reasoning, studies done in Western cultural contexts have shown that, when individuals have power, they tend to show focused, analytic cognitive processing (Guinote, 2007a; Miyamoto & Ji, 2011; P. K. Smith & Trope, 2006). For example, European American participants who were assigned to be a leader on a communication task showed more focused, analytic attention on a subsequent visual task compared with those who were assigned to be a follower (Miyamoto & Wilken, 2010), suggesting that having power increases analytic perception among European Americans. In contrast, in the same study, Japanese participants who were assigned to be a leader showed as much holistic attention as those who were assigned to be a follower; thus, Japanese participants exhibited holistic attention regardless of the assigned position.

Evidence on the cognitive correlates of SES is also accumulating. Studies done in the United States and Europe have repeatedly demonstrated that individuals with higher SES background tend to have analytic cognitive styles compared with those having lower SES background (Grossmann & Varnum, 2011; Kraus et al., 2009; Miyamoto & Ji, 2011). For example, a large survey conducted in the United States found that when presented with a set of three objects, such as *seagull*, *sky*, and *dog*, individuals with higher SES were more likely to group objects based on shared features (e.g., group *seagull* and *dog* because both are animals; taxonomic categorization) rather than based on relationships between the objects (e.g., group *seagull* and *sky* because seagulls fly in the sky; thematic categorization), thus indicating more analytic cognition among high SES Americans (Miyamoto & Ji, 2011; also Na et al., 2010). However, large data collected in China suggest that such a link between higher SES background and taxonomic categorization is weaker in China and especially weak or absent in rice-farming regions (compared with wheat-farming regions). Indeed, in rice-farming region, Chinese participants with higher SES backgrounds were as likely as those with lower SES background to make thematic categorization (Zhang et al., 2021).^3^

#### Summary—Summary Individual Processes

In this section, we reviewed evidence speaking to the idea that another important way in which cultural contexts influence social hierarchy is through individual psychological processes. The evidence suggests that high-ranking individuals in Western cultural contexts tend to be self-oriented and analytic, whereas high-ranking individuals in East Asian cultural contexts tend to be both self-oriented and other-oriented and holistic.

## Discussion and Future Directions

Social hierarchy does not exist in a cultural vacuum. Instead, social hierarchy is embedded in and shaped by cultural meaning systems. Culturally shared values, norms, and expectations determine who is bestowed with higher social rank, socialize those who occupy high ranks as to how to act in public and interact with others, and shape the psychology and behavior of high-ranking individuals. Thus, high-ranking individuals are products of their cultural contexts. Once established, the link between cultural context and social hierarchy can operate in a self-sustaining way.

In Western cultural contexts, such as the United States, research shows that higher social rank is bestowed upon individuals who display unique skills and competences. This is also true in East Asian cultural contexts, such as Japan. However, here, higher social rank is also bestowed because individuals display a concern with mutual obligations and affirm interdependence. In other words, individuals exemplifying cultural ideals are more likely to occupy high rank. Cultural variations also influence what is expected of leaders. High-ranking individuals in Western cultural contexts are expected to be competent and influential. Although high-ranking individuals in East Asian cultural contexts are expected to be competent to a certain degree, they are also expected to be warm and responsible. Moreover, cultural variations influence high-ranking individuals’ behavioral and psychological tendencies. The reviewed research suggests that high-ranking individuals in Western cultural contexts adopt more of a self-oriented perspective. While this is also true for high-ranking individuals in East Asian cultural contexts, the latter additionally adopt more other-oriented perspectives. They adapt their behavior to the situational demands, such that they exhibit behavioral restraint at times but express their anger and even punish group members at other times. Thus, resources associated with high ranks facilitate agency and self-orientation across cultures. But cultural values, norms, and expectations in East Asian cultural contexts seem to orient high-ranking individuals’ action potential to serve interdependence by reducing social conflict and enforcing contributions to the common good.

### Implications of Our Review for Social Hierarchy Research

The reviewed evidence not only summarizes the existing literature on cultural variation between Western and East Asian cultural contexts, but it also clarifies common misinterpretations of these differences when it comes to social hierarchy.

First, our review carries important implications for our understanding of East Asian high-ranking individuals’ actions that can easily be misunderstood. Because of their focus on interpersonal connectedness and interdependence, East Asian cultural contexts are often described as pursuing social harmony (Hashimoto & Yamagishi, 2013). This somewhat romanticized view of East Asian cultural contexts seems less adequate when describing their social hierarchies. For example, psychological other-orientation should not be mistaken for being overly friendly or accommodating. Rather, perspective-taking and empathic concern can yield both positive and negative outcomes (Ku et al., 2015; Longmire & Harrison, 2018; Pierce et al., 2013). Thus, leaders in East Asian cultural contexts have rightfully been described to be both benevolent and authoritarian (Pellegrini & Scandura, 2008). Both tendencies can serve the group’s interdependence and maintain members working together despite strong intragroup vigilance (Liu et al., 2019). Thus, other-orientation of high-ranking individuals in East Asian cultural contexts is consistent with, and at times perhaps necessitates, the expression of anger and dominance to maintain interpersonal collaboration (Kuwabara et al., 2016; J. Park et al., 2013).

Second, high social ranks have been linked to better mental and physical health (Adler et al., 1994; Marmot, 2017). However, cultural variations in expectations associated with high-ranking individuals may have divergent consequences on high-ranking individuals’ mental and physical health across cultures. For example, other-orientation and responsibility expected of high-ranking individuals in Japan may place a certain burden on them. In fact, Japanese managers tend to work long hours, which in turn is linked to higher stress and unhealthy lifestyles (Maruyama & Morimoto, 1996; see also Nakagawa & Yamane, 2018 for the role of emotions and longevity). Indeed, a survey of over 9,000 Japanese workers found that higher occupational status was associated not only with higher eudaimonic well-being but also with worse self-reported physical health (e.g., sleep problems), even when controlling for age and gender (Miyamoto, 2021; see also Park et al., in press). It seems that the responsibility associated with high ranks in Japan may come with both perks and burdens.

Third, because self-orientation is conceptually distinct from other-orientation, high-ranking individuals’ self-orientation does not necessarily need to be accompanied by low levels of other-orientation. One way in which cultural meaning systems might foster high-ranking individuals’ other-oriented alongside self-oriented psychological and behavioral tendencies is through ascribing power with status to them. While power and status are independent concepts in the United States (Blader & Chen, 2012; Blader et al., 2016), research is emerging that in more collectivistic cultures lay theories link power and status (To et al., 2017). This is important because having a sense of power alongside the ability to orient toward others can lead to fairer interpersonal treatment and better decision-making. For example, when powerholders were trained to take another’s perspective, they exhibited more other-oriented behaviors (Galinsky et al., 2014). This suggests that power if combined with status can lead to other-oriented behavior even within Western cultural contexts.

Fourth, dispositional and situational factors have also been shown to moderate the link between high rank and lower other-orientation. For example, individuals who were high on communal orientation made more prosocial choices when they were exposed to a cue in their environment that indicated higher power (S. Chen et al., 2001; Côté et al., 2011). Similarly, high ranks’ feeling of responsibility for others can also influence the link between power and other-orientation (Sassenberg et al., 2012; Scholl et al., 2018). For example, powerholders were more likely to pay attention to the individual information of their subordinates in situations when they had responsibility over them (Overbeck & Park, 2006) and to engage in prosocial behavior toward other team members when power induced feelings of responsibility (Tost & Johnson, 2019). Thus, the link between high rank and decreased other-orientation can be weakened or even reversed among those who have a propensity toward other-orientation even within Western cultural contexts.

### Advancing Previous Theorizing About the Role of Culture and Social Hierarchy

Our review builds on and extends previous theorizing on the impact of power and status across cultural contexts. Geert Hofstede was one of the first social psychologists to observe that cultural contexts systematically differ in the extent to which socio-hierarchical differences are expected and accepted. Surveying more than 100,000 IBM employees in initially 40 countries, Hofstede (2001) found that culture-level scores of responses to questions related to subordinates’ fear of disagreeing with superiors, as well as superiors’ ideal and actual decision-making styles predicted important socio-hierarchical outcomes across families, educational systems, organizations, and even aviation accidents. This dimension, labeled *power distance*, describes the extent to which people rely on their social rank when interacting with others. While East Asian cultures score fairly high on power distance orientation, most Western cultures, including the United States, score fairly low on power distance orientation (Hofstede, 2001). Empirical findings indicate that in high more than in low power distance cultural contexts, people accept and act in accord with their relative hierarchical position (e.g., Brockner et al., 2001). Data from more than 17,000 middle managers in 62 cultures complement these findings and provide additional insights into the relationship between culture and leadership effectiveness (House et al., 2004).

Other scholars have proposed a two-dimensional framework to differentiate cultural contexts that emphasize hierarchy (i.e., vertical cultures) from cultural contexts that value equality (i.e., horizontal cultures; Singelis et al., 1995). Building on Fiske’s theory describing the basic structure (i.e., cognitive schema) of meaningful interactions along four typologies of sociality (A. P. Fiske, 1992), the horizontal–vertical differentiation is situated at the individual level highlighting important differences in cultural orientation (Singelis et al., 1995). Indeed, one important contribution to this theoretical tradition was the development of a scale to assess the four distinct cultural orientations (Triandis & Gelfand, 1998; see also Sivadas et al., 2008). Specifically, while individuals scoring high on vertical collectivism emphasize the integrity of their group and are willing to sacrifice their personal goals for the sake of advancing their group’s goals, individuals scoring high on horizontal collectivism emphasize their similarity with others and the pursuit of common goals. Moreover, individuals scoring high on vertical individualism see themselves as unique by acquiring status and competing with others, whereas individuals scoring high on horizontal individualism see themselves as unique because they are self-reliant (Singelis et al., 1995; Triandis & Gelfand, 1998).

Situating the horizontal–vertical cultural orientation as an individual-level measure has important implications for how individuals may conceptualize and express high rank. For example, Torelli and Shavitt (2010) showed that individuals scoring high on vertical individualism interpreted power in more personalized terms and thus saw power as means for personal advancement (Winter, 1973). In contrast, individuals scoring high on horizontal collectivism interpreted power in more socialized terms and thus saw their power as means for benefiting and helping others (Torelli & Shavitt, 2010). The different conceptualizations of power, in turn, influenced individuals’ thought and behavior. For example, while individuals scoring high in vertical individualism processed information about others in a more stereotypical manner, individuals scoring high in horizontal collectivism processed information in a more individuating manner (Torelli & Shavitt, 2011). Powerholders scoring high on vertical individualism also claimed more resources for themselves (Kopelman, 2009), whereas individuals scoring high on horizontal collectivism, were more likely to exert fairness in their behaviors toward others (To et al., 2020). Thus, the distinction between vertical and horizontal cultural orientations provides important insights into within-cultural nuances of the manifestation of social hierarchy.

Our theoretical model advances prior theorizing about the role of culture in social hierarchy further. It explicitly focuses on both cross-cultural *differences* and *similarities* (see Table 1). For example, our model demonstrates that self-orientation in high-ranking individuals can exist alongside other-orientation. Moreover, it speaks to the *process* through which culture shapes socio-hierarchical relationships and their psychological and behavioral consequences (see Figure 1). It shows how rank differences are embedded in larger cultural meaning systems, which shape how high rank is attained or conferred, and how social hierarchy affords behavior and psychology. The latter, in turn, can reinforce cultural beliefs. It is through a detailed understanding of these processes that we can appreciate the *dynamic nature* of both social hierarchies and the cultural contexts within which they are embedded.

### Advancing Social Hierarchy Research Beyond East Asian and Western/American Cultural Contexts

While most research on social hierarchy taking a cultural perspective has compared U.S. American and East Asian cultural contexts, these comparisons can, of course, only be a stepping stone to more comprehensively study social hierarchy across other cultural contexts.

Indeed, recent research suggests that social hierarchies may differ across collectivistic cultures. For example, South Asian and Arabic cultural contexts are characterized by a more assertive form of interdependence than East Asian cultural contexts (Nishimura et al., 2008; San Martin et al., 2018). Thus, in this respect, South Asian and Arabic cultural contexts seem to resemble the American cultural context, in which leaders are expected to stand up, assert their opinions and signal confidence, as to demonstrate their merit for higher ranks (Ames & Flynn, 2007; Kluegel & Smith, 1986; Sy et al., 2010). Interestingly, research clearly shows that South Asians in the United States are more likely than East Asians in the United States to attain leadership positions, probably because the American cultural context provides a better cultural fit for them (Lu, 2022; Lu et al., 2020). A key explanation for this finding is the important difference in levels of assertiveness between individuals from South Asian versus East Asian cultural contexts, both when measured through self-reports and when measured through peer-ratings (Lu et al., 2022). In contrast, as we reviewed above, in East Asian cultural contexts, leaders are expected to be calm and restraint (Misumi & Peterson, 1985; P. B. Smith et al., 1989; Yamaguchi, 1994). Thus, in Japanese task-oriented groups, the more team leaders exhibited behavioral restraint, the more they were perceived as worthy leaders (Ito et al., 2018). This suggests that individuals are more likely to attain leadership positions the more their traits and behaviors match the cultural prototypes and situational demands (Hais et al., 1997; Hogg et al., 2012; Lord & Maher, 1991), which seem to significantly differ between South Asian and East Asian cultural contexts.

Moreover, Latin American cultural contexts, too, are characterized by a more expressive relational style of interdependence called *simpatia* (Sanchez-Burks et al., 2000; Triandis et al., 1994). As a result, research shows that individuals from Latin American cultural contexts, including Latino-Americans, conceptualize power more in socialized than personalized terms. That is, Latino-Americans think about how having power can be used to achieve goals of the group and not exclusively self-centered goals (Torelli & Shavitt, 2010). Interestingly, powerholders who behaved in a more compassionate way were viewed more positively by Latino Americans, whereas powerholders who behaved in a more equitable manner were viewed more positively by European Americans (Torelli et al., 2015). Thus, Latino-Americans compared to European Americans associated power with more other-orientation both in their conceptualizations of power and the injunctive norms defining how powerholders are expected to behave. Cultural variations between Latino- and European American cultural contexts also exist in how important self- versus other-oriented behaviors are for achieving a higher rank in the workplace (Torelli et al., 2014). In one study, Latino-American participants reported engaging in more warmth-related behaviors, whereas European American participants reported engaging in more competence-related behaviors to achieve a higher rank in their workplace. Warmth-related behaviors were characterized by other-orientation, such as helping others, congratulating them for achievements, and attending a work party. In contrast, competence-related behaviors were characterized by self-orientation, including the display of one’s own awards, attempting to solve tough problems, and signaling confidence (Torelli et al., 2014). It is important to note, however, that while in this respect, Latino American cultural contexts seem to not resemble the European American cultural context, Latin American cultural contexts seem also quite different from East Asian cultural contexts (Krys et al., 2022). Thus, more research is needed to better understand the similarities and differences in social hierarchies across Latin American and East Asian cultural contexts.

At the same time, social psychological research rarely discusses social hierarchies and their impact on interpersonal interactions, as well as on individual thought and behavior for individuals in African cultural contexts. This is a real limitation, as cultural psychological research has shown important ways in which interpersonal relationships (Adams, 2005; S. L. Anderson et al., 2008), social roles (Salter & Adams, 2012), and the experiences of health and illness (Adams & Salter, 2007; Luhrmann et al., 2015) differ between West African, Western European, and American cultural contexts. Moreover, cross-cultural work on management has pointed to emic patterns of managerial leadership in Sub-Saharan African cultural contexts (Wanasika et al., 2011). For example, the idea of *ubuntu*, which is the emphasis on interdependence of humanity and literally means “I am because we are; I can only be a person through others” (Mbigi, 2007, p. 297), has been suggested to be a key value of sub-Saharan African leadership (Mbigi, 2007; Wanasika et al., 2011). In fact, analyses of the aforementioned GLOBE Project (House et al., 2004) found that humane-oriented attributes that include supportive and considerate leader behaviors and compassion/generosity, which resemble *ubuntu*, were perceived to be especially important characteristics for effective leadership in sub-Saharan African cultural contexts (Wanasika et al., 2011). It would thus be important for future social psychological research to examine if collective, interpersonal, and individual processes of high-ranking individuals found in East Asian cultural contexts would also be found in African cultural contexts or if they take different forms.

Finally, recent research suggests that social hierarchies may differ across Western cultures. For example, while SES was significantly negatively associated with the endorsement of other-oriented socialization values in Frontier cultures, including the United States and Australia, the association was not significant in European cultures, including Germany and Spain (Miyamoto et al., 2018). It is possible that the history of voluntary settlement in Frontier cultures have especially contributed to the link between high rank and low other-orientation. Alternatively, or additionally, it is possible that European cultural contexts afford individuals to balance individualism with concern for others (see Gobel et al., 2018). Thus, high-ranking individuals in European cultural contexts are self-centered and yet can behave in an other-oriented and prosocial manner (Schmukle et al., 2019; Stamos et al., 2019; von Hermanni & Tutić, 2019). For example, experiments have shown that if afforded to make intergenerational decisions, powerholders experience a greater sense of responsibility (Tost et al., 2015). Powerholders’ feeling of responsibility for others, in turn, increased their other-orientated tendencies (Sassenberg et al., 2012; Scholl et al., 2018). We think that both hypotheses as to why social hierarchies may differ across Western cultures warrant further examination.

### Future Research Questions

Our conceptual framework sheds a new light on existing findings and proposes new questions worth testing in the future (Table 2). One question for future research is to explore the distal and proximal origins of the cultural variations in social hierarchies. For example, cultural psychologists have started to examine the importance of socio-ecologies (Talhelm et al., 2014; Uchida et al., 2019; Uskul et al., 2008; Uskul & Oishi, 2018) for explaining the functional value of cultural variations. A question for future research is what the role of a leader in different ecologies, such as rice farming, wheat farming, fishing, or herding might be. Beyond these distal factors, it would also be informative to explore more proximal factors that give rise to and maintain cultural variations in responsibility and other-orientation among high-ranking individuals. In Japan, for example, high- and low-ranking individuals are expected to socialize after work as part of *nomikai*. These drinking parties afford low-ranking individuals a social context to articulate concerns and complaints without challenging the existing social hierarchy at work. In Western cultural contexts, daily interactions between high- and low-ranking individuals can include the signaling of social ranks and affirming the status quo, as suggested by studies of thin slices of behavior and speech (e.g., Kraus et al., 2019; Kraus & Keltner, 2009). Thus, future research is needed to improve our understanding of the origins and maintenance of social hierarchy across cultural contexts through mutual constitution of meaning-systems and structure.

**Table 2.** Outstanding Questions.

*Questions raised by a cultural psychological approach to social hierarchy*
1. What are the distal and proximal origins of the cultural variations in social hierarchies? And how is the status quo maintained in different cultural contexts? 2. What are the psychological manifestations of high social rank in non- East Asian collectivistic cultures (e.g., South Asia, Middle East, or Latin America)? And what can we learn about (and from) social hierarchy(ies) in African cultural contexts? 3. What is the role of achieved versus ascribed status characteristics for occupying high social rank across cultural contexts? And how do these different bases of social rank shape psychological and behavioral manifestations of self- and other-orientation? 4. What is the meaning and function of meritocracy across cultures? And how does it interact or is reconciled with the other-oriented tendencies of high-ranking individuals in East Asian cultural contexts? 5. What dispositions and situations afford high-ranking individuals in Western cultural contexts to be more other-oriented? 6. What are the consequences of self- versus other-orientation for high-ranking individuals’ mental well-being and health? 7. How do dynamic changes to culture affect the self- and other-oriented tendencies of high-ranking individuals? 8. How does culture interact with economic inequality to shape self- and other-orientation in high-ranking individuals?

We think that one important area for future research is the role of achieved versus ascribed status characteristics across cultural contexts. As mentioned before, sociologists have long acknowledged that rank orders can be based on different status characteristics (e.g., Berger et al., 1972). Past research investigating the link between culture and social hierarchy found that achieved status characteristics are likely a meaningful basis of social status in most cultures (e.g., House et al., 2004). Ascribed status characteristics, such as age, sex, or familial class background, which are determined through birth and therefore fairly stable, may differ more widely in their importance across cultures. For example, age is a very important component of social ranking in East Asian cultural contexts (Sung, 2001), and recent research found that business and political leaders were significantly older in East Asian compared to Western cultural contexts (Vaughan-Johnston et al., 2021). Moreover, individuals from the French cultural context acknowledged that familial class background was a more important basis for occupying high rank compared to individuals from an American cultural context (Gobel & Kim, 2023). Consistent with these findings, individuals from Mediterranean European cultural contexts, such as Italy or France, compared to individuals from the American cultural context, also reported perceiving less intergenerational mobility (Alesina et al., 2018). Thus, it seems plausible that ascribed status characteristics would play an important role in shaping the effects of social hierarchies across Mediterranean European, East Asian, South Asian and Latin American cultural contexts.

Future research is also needed to better understand the interplay of seemingly opposing cultural beliefs for the functioning of social hierarchy. For example, meritocratic beliefs are widespread across Western cultural contexts (Mijs, 2021; Reynolds & Xian, 2014). At the same time, even in East Asian cultural contexts, where the importance of diligence and achievement has been emphasized in the Confucian teaching (Bond & Hwang, 1986), merit-based differences are an important aspect of social hierarchies (Li & Hu, 2021; Oishi et al., 2022). In ancient China, for example, the imperial examination system ensured selecting of a political and economic elite based on their performance (Xie, 2016), and such merit-based social hierarchies have seen a renaissance as part of China’s recent economic development (Bian, 2002; Song, 2009). Similarly, post-war Japan and South Korea were characterized by strong beliefs that individual effort and hard work ensured personal prosperity (Chiavacci, 2008). While East Asian models of “middle-class societies” have been threatened by the economic crises in the 1990s and the emergence of dual labor markets, meritocratic principles remain a characterizing feature of East Asian cultural contexts (Chiavacci & Hommerich, 2016). Future research is needed to better understand the meaning and function of merit (e.g., innate ability vs. effort) across cultural contexts as well as how the cultural tradition of meritocracy influences the other-oriented psychological tendencies of high-ranking individuals this review described.

We think that another important area for future research is the investigation of how dynamic changes to culture affect the self- and other-oriented tendencies of high-ranking individuals. As we said before, any reification of culture would be mistaken. Instead cultures change (Kashima et al., 2019). Indeed, recent advances in cultural evolution elucidate the micro-level mechanisms, such as social learning, that potentially shape cultural information over time (e.g., Kashima, 2016; Mesoudi, 2009). At the macro-level, research suggests that individualistic cultural contexts, such as the United States, are becoming more individualistic (Hamamura, 2012). For example, Americans report increasing levels of self-esteem (Twenge et al., 2017), and they are becoming more narcissistic (Twenge et al., 2008). At the same time, divorce rates are up, while household size is down, and the number of multigenerational households is declining (Grossmann & Varnum, 2015). Interestingly, similar developments are taking place in other parts of the world, including in East Asian cultural contexts such as China and Japan (Inglehart & Baker, 2000; Santos et al., 2017). Here, too, self-directional values are being endorsed more (Xu & Hamamura, 2014; Zeng & Greenfield, 2015), and the family structure is changing (Ogihara, 2018; Santos et al., 2017). Thus, one possibility is that through globalization, the Westernization of the world will reduce cultural differences in the other-orientation of high-ranking individuals over time. Alternatively, and in line with the argument put forward in this review, cultural contexts may continue to shape the organization of economies and the expression of capitalism across different societies contributing to an active reinvention and reincorporation of non-Western cultural patterns (Hamamura, 2012, 2020).

Another important aspect of cultural change is linked to rapidly increasing levels of economic inequality in a globalizing world. Economic inequality provides one of the most important contextual influences on human psychology in the present (Rodríguez-Bailón et al., 2020; Willis et al., 2022). For example, greater economic inequality results in greater self-orientation, increases the psychological distance to others, and leads to greater competition (Petkanopoulou et al., 2018; Sánchez-Rodríguez et al., 2019; Sommet et al., 2019). Research has also shown that greater economic inequality alters individuals’ perception of how deserving others are (Heiserman & Simpson, 2017). Moreover, research conducted in very unequal contexts, such as the United States, demonstrates that generalized trust and confidence in government is declining (Hamamura, 2012; Twenge et al., 2014), and citizen’s confidence in democracy is shattered (Foa & Mounk, 2016; Inglehart, 2016). Yet, as with the literature on social hierarchy reviewed above, these findings are mostly originating from Western cultural contexts, and initial research in East Asian cultural contexts suggest that economic inequality in East Asia is unrelated to the erosion of social cohesion (Delhey et al., 2018). In fact, in East Asian cultural contexts, which score high on power distance beliefs, some form of inequality may be expected and accepted (Hofstede, 2001). This raises the question whether some form of inequality may even be required for high-ranking individuals in East Asian cultural contexts to effectively fulfill their social role. Thus, investigating the link between the psychological effects of social hierarchy and structural effects of economic inequality across cultural contexts is an important task for future research.

## Conclusion

We provided an integrative review of the current state of social hierarchy research from a cultural psychological perspective comparing Western (mostly American) and East Asian cultural contexts. Such a cultural psychological approach revealed that high-ranking individuals across cultural contexts were characterized by high levels of self-orientation and agency. However, their emotional, cognitive, and behavioral other-orientation differed dramatically across cultural contexts. Specifically, high-ranking individuals in East Asian cultural contexts were more other-oriented on all of these dimensions, compared to their counterparts from Western cultural contexts. In fact, an analysis of the accompanying socialization practices and collective expectations showed that they were socialized for and indeed selected because of other-oriented interpersonal and individual tendencies. Living up to these expectations, in turn, seemed to reinforce socio-hierarchical differences in East Asian cultural contexts. Thus, this review provides an important illustration of how cultural contexts and manifestations of social hierarchy mutually constitute each other. We urge that more work examines social hierarchy and its manifestations across the globe.

## Constraints on Generality Statement

As is often the case in social psychology, studies investigating how social hierarchy shapes behavioral and psychological processes are frequently based on university student samples. Throughout this review, we have tried to point out where this was the case. At the same time, research on social hierarchy also includes quite a few studies collecting data in organizations using non-student samples. In our view, the biggest limitation of this literature, and indeed the inspiration for this review article, is that the social hierarchy literature to-date is often limited by generalizations based on findings from Western/American samples. With this review paper we try to build a stepping stone so that future research can change this.

## Positional Statement

The first author was socialized in a Western cultural context, and the senior author was socialized in an East Asian cultural context. Both authors have lived, taught, and conducted research in Western cultural contexts, such as the United States, and East Asian cultural contexts, such as Japan. Our approach to writing this review was to even-handedly and systematically describe the existing literature and to point out where we saw important gaps in this literature.

## Notes

1. In this review, for the sake of brevity, we sometimes write “East Asians” or “European Americans,” but it is important to note that these terms refer to people who participate in the artifacts, practices, and institutions prevalent in East Asian (or European American) cultural contexts.

2. Self- and other-orientations refer to specific psychological processes and behaviors of individuals that support and promote the self or others. Self- and other-orientations fit and affirm independent and interdependent models of the self, respectively. At the same time, this does not necessarily mean that other-orientation (self-orientation) is incompatible with an independent (interdependent) model of the self. Other-orientation can sometimes serve an independent model of the self (e.g., when donating to and volunteering for causes that support individualistic values; Kemmelmeier et al., 2006), and self-orientation can sometimes serve an interdependent model of the self (e.g., when academic achievement is an obligation to parents and others; Tao & Hong, 2014). We believe that such a nature of self- and other-orientation is especially useful in understanding manifestations of social hierarchy across cultures.

3. Interestingly, Grossmann and Varnum (2011) found that college students from higher SES backgrounds are more analytic than those from lower SES backgrounds across both the U.S. and Russia, suggesting that the meaning systems surrounding hierarchy in Confucian cultures may not necessarily generalize to other types of collectivistic cultures.
